# Nanoscale electrical properties of epitaxial Cu_3_Ge film

**DOI:** 10.1038/srep28818

**Published:** 2016-07-01

**Authors:** Fan Wu, Wei Cai, Jia Gao, Yueh-Lin Loo, Nan Yao

**Affiliations:** 1 Princeton Institute for the Science and Technology of Materials, Princeton University, 70 Prospect Avenue, Princeton, New Jersey 08544, USA

## Abstract

Cu_3_Ge has been pursued as next-generation interconnection/contact material due to its high thermal stability, low bulk resistivity and diffusion barrier property. Improvements in electrical performance and structure of Cu_3_Ge have attracted great attention in the past decades. Despite the remarkable progress in Cu_3_Ge fabrication on various substrates by different deposition methods, polycrystalline films with excess Ge were frequently obtained. Moreover, the characterization of nanoscale electrical properties remains challenging. Here we show the fabrication of epitaxial Cu_3_Ge thin film and its nanoscale electrical properties, which are directly correlated with localized film microstructures and supported by HRTEM observations. The average resistivity and work function of epitaxial Cu_3_Ge thin film are measured to be 6 ± 1 μΩ cm and ~4.47 ± 0.02 eV respectively, qualifying it as a good alternative to Cu.

Cu_3_Ge is one of the best alternatives to Cu for contacts/interconnections in microelectronics industry, owing to its thermal stability up to 450 °C^1^ and low bulk resistivity (~8 ± 2 μΩ cm) over a large Ge composition range of 25–35 at.%^1^,^2^. More importantly, the out diffusion of Cu is suppressed^3^, such that diffusion barrier is no longer needed and the service life of interconnects can be improved considerably. In addition, Cu_3_Ge remains stable against oxidation in air up to ~500 °C^3^,^4^. Consequently, Cu_3_Ge has been considered superior to Cu as contacts/ interconnections for integrated circuit devices.

Cu_3_Ge has been reported to grow on various substrates, including Si^3^,^5^,^6^,^7^, Si_x_Ge_1−x_^5^,^6^,^8^, Ge^7^,^9^, GaAs^10^,^11^,^12^, GaN^13^,YBa_2_Cu_3_O_7−x_^14^, Ta/TaN^15^. Different deposition methods have been explored to fabricate Cu_3_Ge thin films, such as sputtering/ electron beam^1^,^2^,^3^,^9^,^14^,^16^, thermal evaporation^17^, and vapor–solid reaction^15^. Despite the remarkable progress, the reported Cu_3_Ge films were frequently polycrystalline with excess Ge. Epitaxial Cu_3_Ge film is highly desired owing to reduced diffusion paths (grain boundaries) and possibly lower electrical resistivity. Therefore the natural question to ask is: can epitaxial Cu_3_Ge thin film be fabricated and what are its electrical properties?

In this paper, epitaxial Cu_3_Ge thin film is fabricated and its nanoscale electrical properties are characterized by conductive atomic force microscopy (CAFM) and Kelvin probe force microscopy (KPFM). As integrated device structures continue to shrink in size, the change in electrical properties needs to be characterized on nanoscale^18^ too. Conventional electrical characterization approaches are limited by lithographic dimensions and probe positioning^19^, while CAFM and KPFM are capable of probing electrical characteristics with nanoscale resolution^20^. Moreover, CAFM and KPFM enable simultaneous mapping of nanoscale structure and electrical properties at the same sample location^21^. Here, the nanoscale electrical properties of epitaxial Cu_3_Ge thin film are found to be governed by localized film morphologies, and the resistance between two points on the film is proportional to their distance. The average resistivity of epitaxial Cu_3_Ge thin film is measured to be ~6 ± 1 μΩcm, 20% smaller than the average value (8 ± 2 μΩcm) reported for polycrystalline Cu_3_Ge films^22^,^23^. The average work function of epitaxial Cu_3_Ge thin film is measured to be ~4.47 ± 0.02 eV, rendering it a desirable mid-gap gate metal to be used directly over SiO_2_ for CMOS devices because it requires minimal and symmetric channel implants even at linewidths below 0.5 μm^24^. Since the application of sapphire substrate is limited, the fabrication parameters and characterization methods reported here can be used as future reference for fabrication and characterization of epitaxial Cu_3_Ge films integrated on other substrates.

## Results and Discussions

**Figure 1:**
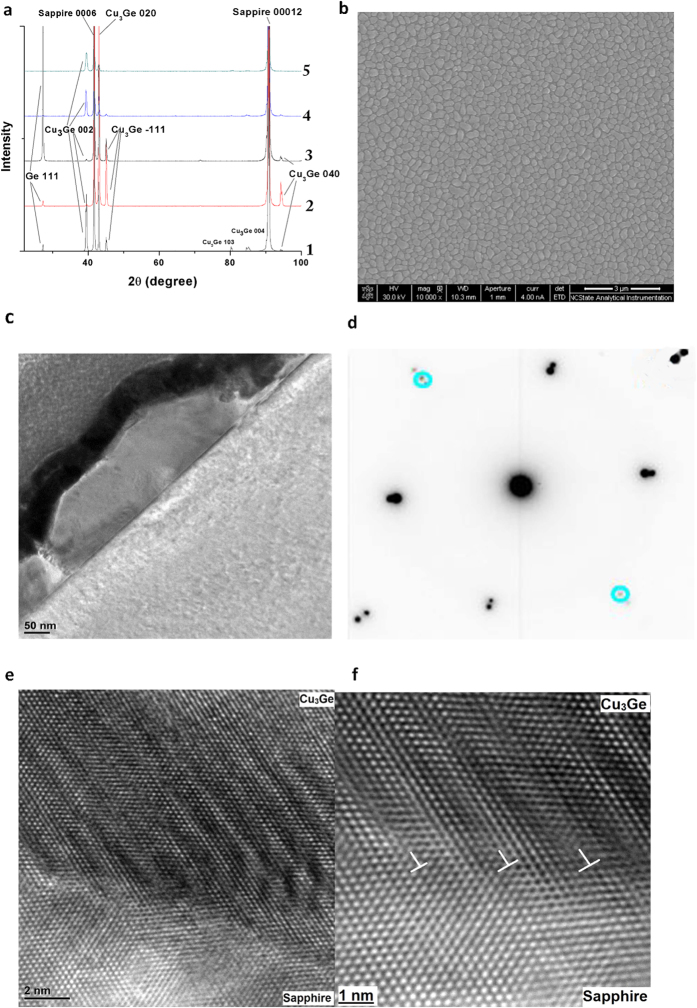
Structural characterization of Cu_3_Ge thin films. (**a)** XRD θ–2θ patterns of sample 1 to 5. (**b)** Typical SEM image of the epitaxial Cu_3_Ge thin film (sample 5). (**c)** Typical bright-field TEM image of epitaxial Cu_3_Ge thin film. (**d)** Diffraction pattern of the Cu_3_Ge/c-sapphire interface. (**e)** HRTEM image of the Cu_3_Ge/c-sapphire interface. (**f)** Enlarged HRTEM image of Cu_3_Ge/c-sapphire interface.

In order to obtain epitaxial Cu_3_Ge thin films, a series of (5) samples were fabricated on c-sapphire substrate by controlling the deposition parameters of pulsed laser deposition (PLD). Specifically, 90 thin layers of Cu and Ge (thus a total of 180 layers) were deposited alternatively on c-sapphire for each sample. The laser pulse numbers in each repetition of Cu and Ge ablations from sample 1 to 5 are 35:5, 25:5, 15:5, 14:2 and 7:1 (see supplementary information, section 1 for details). Figure 1a demonstrates the XRD θ–2θ patterns of 5 samples, in which the peaks are labelled. Ge (111) peak (@27.32°) appears for sample 1–3, indicating the existence of excess Ge. In contrast, pure Cu_3_Ge thin films were obtained for samples 4 and 5, as evidenced by the disappearance of Ge peak. However, the obtained Cu_3_Ge thin film is still polycrystalline for sample 4, as shown by the Cu_3_Ge {002}, {020}, {−111} peaks. Only Cu_3_Ge {002} and {020} peaks exist in the XRD θ–2θ pattern of sample 5, indicating a bi-epitaxial relationship between the Cu_3_Ge thin film and the c-sapphire substrate. Since only 1 laser pulse was used to ablate each Ge layer for sample 5, this should be the optimum condition for epitaxial Cu_3_Ge thin film fabrication by PLD. Hereafter, the detailed microstructural and electrical characterizations will be focused on sample 5, whose Cu_3_Ge thin film has the best crystallinity and an epitaxial relationship with the c-sapphire substrate.

Figure 1b is the plan-view SEM image, showing a uniform Cu_3_Ge film without chunks or Ge grains. Cu_3_Ge islands with similar sizes distribute uniformly on the substrate, with grain boundaries around the islands. Figure 1c is a bright-field cross-section TEM image, showing the typical microstructure of the epitaxial film. The Pt layer was deposited during FIB sample preparation for protection. The Cu_3_Ge growing on c-sapphire has an average height of ~150 ± 10 nm. The interface between Cu_3_Ge and c-sapphire is sharp and straight, without traces of diffusion and secondary phases. To study the orientation relationships between epitaxial Cu_3_Ge film and c-sapphire, selected area diffraction pattern (SADP) was obtained at the interface and shown in Fig. 1d. The SADP demonstrates an epitaxial relationship between Cu_3_Ge film and c-sapphire substrate. Though the low-order diffraction points lie close to or even overlap with each other, the higher-order diffraction points split and appear along the same directions. Some diffraction points of c-sapphire are labelled with light blue circles, while the other diffraction points are not labelled on purpose for clear visualization.

To investigate the atomic structure at the interface region, HRTEM images were collected and shown in Fig. 1e. The viewing direction is [110] zone of c-sapphire. Periodic contrast is visible along the interface due to the existence of misfit dislocations with a certain separation, indicating that the Cu_3_Ge island/c-sapphire interface is semicoherent^25^. Periodic dislocations exist at the interface to accommodate the misfit strain between two phases. For c-sapphire and Cu_3_Ge, the interface planes are (001) and (010), respectively. Both of them share a rectangular lattice (width = 4.759 Å, length = 8.243 Å for (001) sapphire; width = 4.54 Å, length = 4.22 Å for (010) Cu_3_Ge). Therefore, one lattice of the c-sapphire (001) plane matches with 2 lattices of Cu_3_Ge (010) plane. The lattice misfit of the width between c-sapphire (001) and Cu_3_Ge (010) is ~4.60%, and the lattice misfit of the length between one sapphire (001) and two Cu_3_Ge (010) is ~−2.33%. To further study the detailed atomic structure, the interface region is enlarged for HRTEM observation and shown in Fig. 1f. The Cu_3_Ge planes connect with the lattice planes of c-sapphire across the interface between the interfacial dislocations and bend only within the localized width of the misfit dislocations. Arrangement of misfit dislocations at the Cu_3_Ge/c-sapphire interface is evident and labelled in Fig. 1f. More structural investigations and discussions can be found in our previous work^26^.

**Figure 2:**
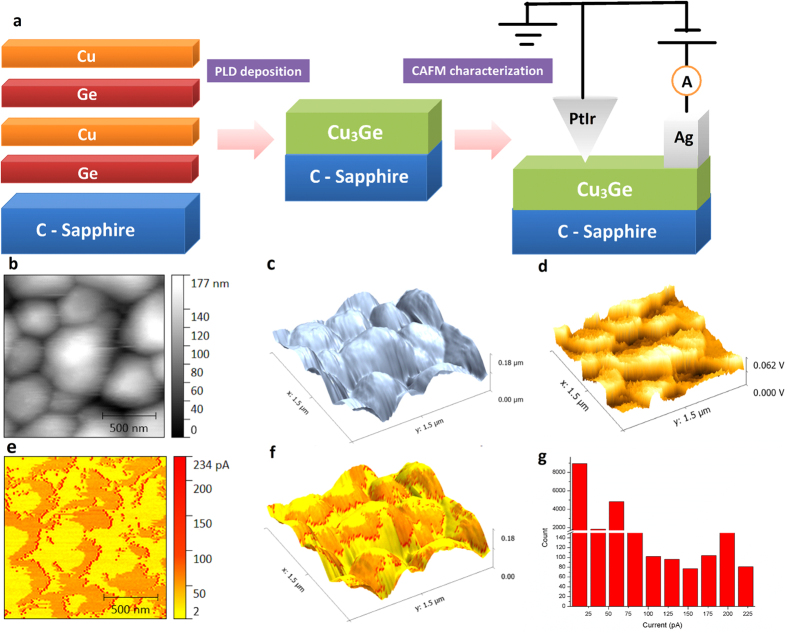
CAFM characterization of epitaxial Cu_3_Ge thin film. (**a)** Schematic illustration of the CAFM characterization setup. (**b–f)** 2-D, 3-D AFM height image, deflection image and 2-D, 3-D current map of the epitaxial Cu_3_Ge thin film. (**e)** Current histogram: a quantitative summary of the electron transport capabilities of the epitaxial Cu_3_Ge film.

As integrated device structures approach the nanometer scale, the change in electrical properties needs to be characterized on the same scale^18^, where conventional bulk electrical characterization approaches are limited by lithographic dimensions and probe positioning^19^. As demanded by the highly integrated device structures, the localized nanoscale electrical properties of a film surface need to be measured on specific contacts or locations, providing diagnostic data to determine the failure mechanism, find the location of faults, and characterize defects of devices with microleakages or higher contact resistance^27^. Therefore it is the nanoscale electrical properties of a film that determines the performance of device structures/contacts/interconnects. The nanoscale electrical characterization by CAFM of the PLD-fabricated epitaxial Cu_3_Ge thin film is schematically illustrated in Fig. 2a. A thin uniform layer (3 mm × 1 mm) of Ag was applied on one end of the film as a fixed electrode, while the Pt/Ir -coated AFM tip functioned as a movable electrode with nanometer-scale precision in position and a controlled nanonewton-range force^28^. The conductive tip enables simultaneous acquisition of topographic/current images, current-voltage trace^20^ and also an easy contact with various substances without complex lithography processes^18^. The simultaneously obtained 3D AFM height image, deflection image and current map of the epitaxial Cu_3_Ge thin film are displayed in Fig. 2b–f. The height images (Fig. 2b,c) provide quantitative measurements of surface roughness, surface features and thickness, while the deflection image (Fig. 2d) presents higher resolution of surface features owing to better frequency response^29^. The scanned area shown in Fig. 2b–d is 1.5 μm × 1.5 μm. The average diameter of Cu_3_Ge islands is demonstrated to be ~300–600 nm, with island heights ranging from 50 to 180 nm. As the Pt/Ir-coated AFM tip scanned over the sample surface with a constant force and an applied voltage, the local current was recorded as a function of positions to provide a map showing the local capabilities to transport charge. The resulting current map (Fig. 2e) of epitaxial Cu_3_Ge film was obtained from the same region of Fig. 2b and overlaid onto the latter to form Fig. 2f. The color (from yellow to red) represents the measured local currents. Figure 2f shows that the majority of current bursts (red-color region) are neither on island tops nor in the valleys, but across the hillside. Therefore the nanoscale electrical conductivities are highly relevant to the localized film morphology. The current values at different spots of the film are extracted to produce the current histogram (Fig. 2g), summarizing the electron transport capabilities of the epitaxial Cu_3_Ge film. From Fig. 2g, it is evident that a large fraction of the surface area has a small capability of electron transport, owing to the discontinuous current paths resulting from disconnected island structure. For the current range of 75 pA to 225 pA, the counts for different currents are close, indicating a uniform distribution of highly and less conductive areas.

**Figure 3:**
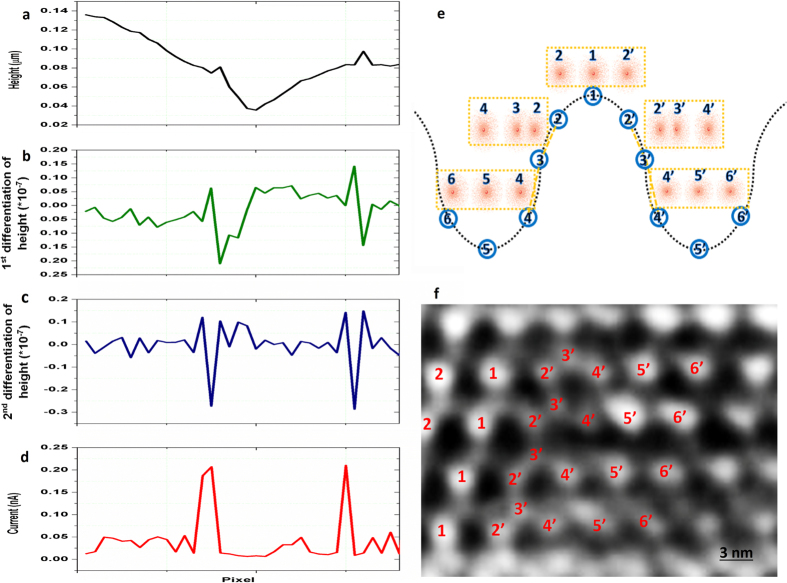
Current line-scan profile analysis of epitaxial Cu_3_Ge thin film. **(a)** A typical line profile derived from the height image (Fig. 2b). (**b–c)** The 1st and 2nd differentiations of the height line profile derived from (**a)**. (**d)** Corresponding electrical current line profile derived from the current map. (**e)** The schematic illustration of atoms on film surface with changing atomic distances. (**f)** HRTEM image of the area with current burst, the atoms are labelled accordingly and can match well with the schematic illustration in (**e**).

To correlate the localized electron transport capability with film morphology in a clearer manner, line-scan profile analysis was performed on the morphological and current maps. Figure 3a shows a typical line profile derived from the height image. The corresponding line profile of electrical current is derived from the current map and demonstrated in Fig. 3d. The 1^st^ and 2^nd^ differentiations of the height line profile were derived and plotted in Fig. 3b,c for better analyses. Comparison between Fig. 3b,d reveals that current burst generally occurs at a position where height change rate reaches a local maximum. This phenomenon seems to be ubiquitous for our film, as supported by randomly derived line-scan profiles (see supplementary information section 2, Figures S1–S3). From the atomic-scale perspective, the localized height change corresponds directly to the atomic distance. If the height change rate is relatively constant over a region, the atomic distances among different atoms remain constant. Nevertheless, if the height change rate reaches a local maximum, the atomic distances change correspondingly. Such scenario is schematically illustrated in Fig. 3e, where the dashed wavy line represents the surface morphology and blue circles stand for atoms on the surface. The atomic distance between adjacent atoms remains the same for each atom in this illustration, except atoms 3 and 3′. The free electron densities around different atomic nuclei were schematically drawn in the yellow dashed rectangles. As the atomic distance between atoms 2 and 3 decreases, the free electron density is accordingly larger than that between other atoms. Since electrical current in metals is directly proportional to the free electron density, a localized electrical current burst will occur around atoms 2 and 3, whose atomic distance/ height change rate is the local minimum. To prove this correlation mechanism, we performed HRTEM observation of the area with current burst by using FIB to prepare TEM samples. The atoms in Fig. 3f are labelled accordingly and can match well with the schematic illustration in Fig. 3e. It proves that the atomic distances are not constant in the current burst region, resulting to the local maximum electron density. This phenomenon was well-known as the strain-current relationship and supported by others^30^. It is worth to note that the electron transfer capability from the sample to the tip may also change as the tip is scanning over the film, thus contributing to the current burst observed.

**Figure 4:**
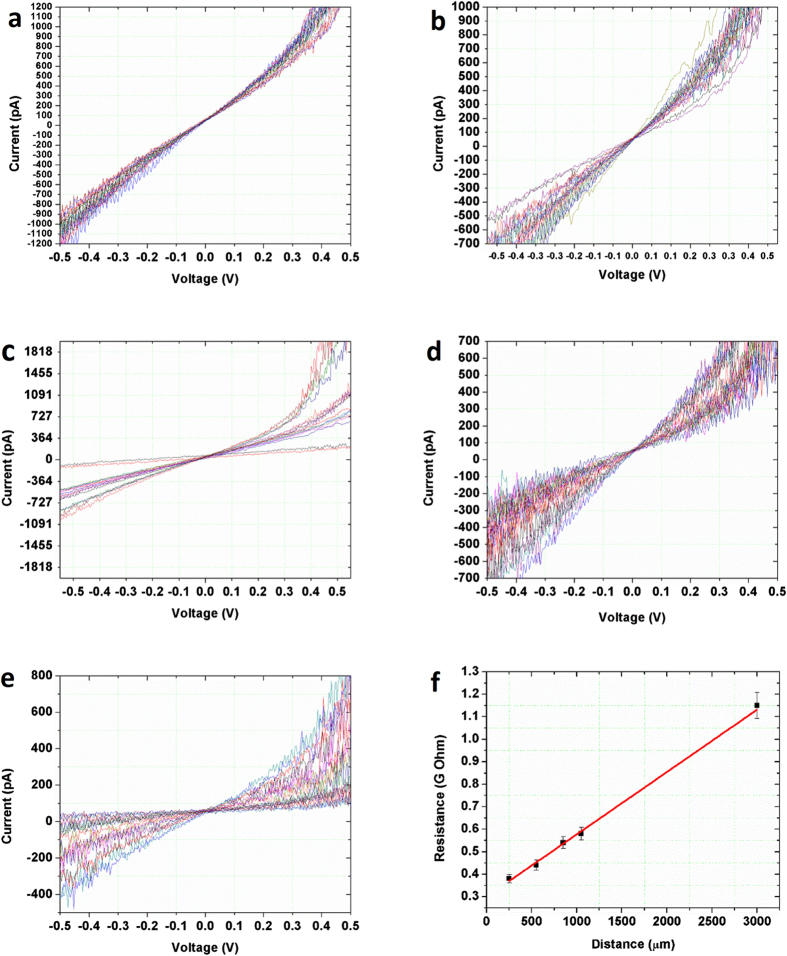
Current-voltage (I-V) curves obtained by changing the distance between the Pt/Ir electrode and the Ag electrode. From (**a**–**e)**, the distances between two electrodes are 250 μm, 550 μm, 850 μm, 1050 μm and 3000 μm, respectively. (**f)** The average total resistance calculated from the five groups of I-V curves (**a–e**) as a function of the distance between two electrodes.

Although the current map can reflect the localized electron transportation capabilities, dividing the applied voltage by the measured current only generates the total resistance (R_t_), which includes contact resistance (R_c_), internal system resistance (R_i_), and sample resistance (R_s_). To account for R_c_ and R_i_, the transmission line method (TLM)^31^ was employed, in which the total resistance R_t_ was measured as a function of the distance between the mobile Pt/Ir electrode and the immobile Ag electrode. Since the applied force during the electrical measurement remains constant, the sum of R_c_ and R_i_ will remain unchanged and can be extracted from the y-intercept. R_s_ can thus sequentially be obtained by R_s_ = R_t_ − R_i_ − R_c_. In our case, local current–voltage curves were collected by placing the tip at 5 groups of different positions and varying the applied bias from −500 mV to +500 mV. In each group, 16 I-V measurements were performed at different positions locating approximately at the same distance away from the Ag electrode. By averaging the 16 R_t_s calculated from the 16 I-V curves in each group, the total resistance (R_t_) at a specific distance is obtained. Figure 4a–e demonstrate the 5 groups of I-V curves (16 in each). The distances between the Pt/Ir and Ag electrode from Fig. 4a–e are 250 μm, 550 μm, 850 μm, 1050 μm and 3000 μm, respectively. The majority of the I-V curves show linear responses, indicating ohmic electrical transport. Some I-V curves show a non-linear behavior, owing to the unstable complex tip-sample nano-contact, especially when the applied force is small.

The average total resistance (R_t_) calculated from the five groups of I-V curves were plotted as a function of the distance between two electrodes, as shown in Fig. 4f. It shows that the measured total resistance (R_t_) changes linearly with the distance between two electrodes. A straight line was added to fit the data points for better analysis, resulting to a linear function R_t_ = 0.31 GΩ +2.7 × 10^−4^ (GΩ/μm) × distance (μm). Therefore the sum of R_c_ and R_i_ equals the y-intercept (i.e. 0.31 GΩ), and the measured Cu_3_Ge thin film resistance (R_s_) is linearly relevant to the distance: R_s_ = ρl/A, where l is the distance between two electrodes, A is the cross-sectional area for current to go through, and ρ is the resistivity of epitaxial Cu_3_Ge thin film. The average resistivity of epitaxial Cu_3_Ge thin film can be calculated as ~6 ± 1 μΩ cm (see supplementary information, section 3 for detailed calculations), which is 20% smaller than the average value (8 ± 2 μΩcm) reported for polycrystalline Cu_3_Ge films^22^,^23^.

**Figure 5:**
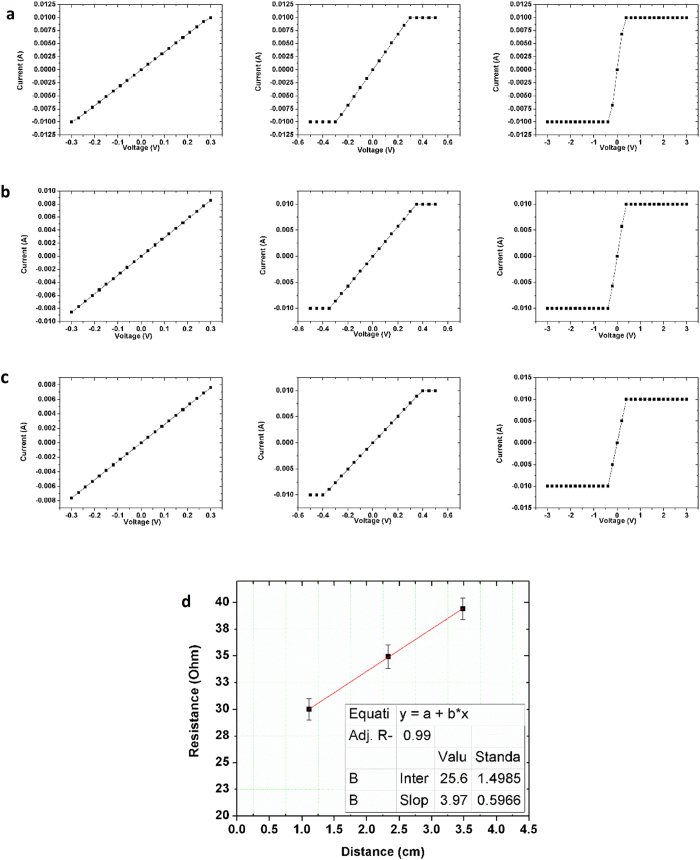
Bulk electrical measurement of epitaxial Cu_3_Ge thin film. **(a–c)** 9 I-V measurements performed at a distance of 1.11 cm, 2.33 cm and 3.48 cm between two probes by varying the input voltage, from left to right: −300 mV to 300 mV; −500 mV to 500 mV; and −3000 mV to 3000 mV. **(d)** The plot of total resistance as a function of the distance between two probes.

To corroborate that the resistivity obtained by nanoscale electrical measurement was correct, we also performed bulk resistivity measurement on our sample. The total resistance R_t_ was measured as a function of the distance between two probes of the Lakeshore probe system (see Methods for more details). Three different probe distances (1.11 cm, 2.33 cm and 3.48 cm) were selected for our bulk measurement. At each specific distance, 3 different I-V measurements were performed by varying the input voltage: (1) from −300 mV to 300 mV; (2) from −500 mV to 500 mV; (3) from −3000 mV to 3000 mV. The total 9 I-V curves were plotted in Fig. 5a–c. The current limit of the Agilent 4155C semiconductor parameter analyzer was set at +10 mA for protection, such that any current larger than 10 mA was cut off in the diagrams. All 9 diagrams show a linear relationship between current and voltage, demonstrating that the epitaxial Cu_3_Ge film is macroscopically conductive. As the distance between two probes increased, the current measured at the same applied voltage decreased, indicating a larger resistance at a longer probe distance. The total resistance(R_t_) for the probe distances of 1.11 cm, 2.33 cm and 3.48 cm were measured from Fig. 5 to be ~30.0 Ω, ~34.9 Ω and ~39.4 Ω respectively. The plot of total resistance(Rt) as a function of the distance between two probes is shown in Fig. 5d. By changing the distance between two probes while maintaining all the other parameters, such as the input current and contact conditions, the sum of R_c_ and R_i_ will remain unchanged, and only R_s_ changes with the probe distance. Therefore the sum of R_c_ and R_i_ can be extracted from the y-intercept. As shown in Fig. 5d, R_t_ changes linearly with the distance between two probes, and a straight line was added to fit the data points for better analysis, resulting to a linear function of R_t_ = 25.6 Ω +3.97 (Ω/cm) × distance (cm). Therefore the sum of R_c_ and R_i_ equals the y-intercept (i.e. 25.6 Ω), and the measured Cu_3_Ge thin film resistance (R_s_) is linearly relevant to the distance: Rs = ρl/A, where l is the distance between two probes, A is the cross-sectional area for current to go through, and ρ is the resistivity of epitaxial Cu_3_Ge thin film. Since the average thickness of the film is ~150 nm, and the width for current to go through the film is ~1 mm, the average resistivity of epitaxial Cu_3_Ge thin film can be calculated as ρ = 3.97 (Ω/cm) × 1 mm × 150 nm = 5.96 (μΩ cm), which is close to the value obtained by nanoscale measurement. Considering that diffusion barrier is no longer needed for Cu_3_Ge to replace Cu, the overall resistivity of epitaxial Cu_3_Ge thin film qualifies its application as the new-generation interconnection material.

**Figure 6:**
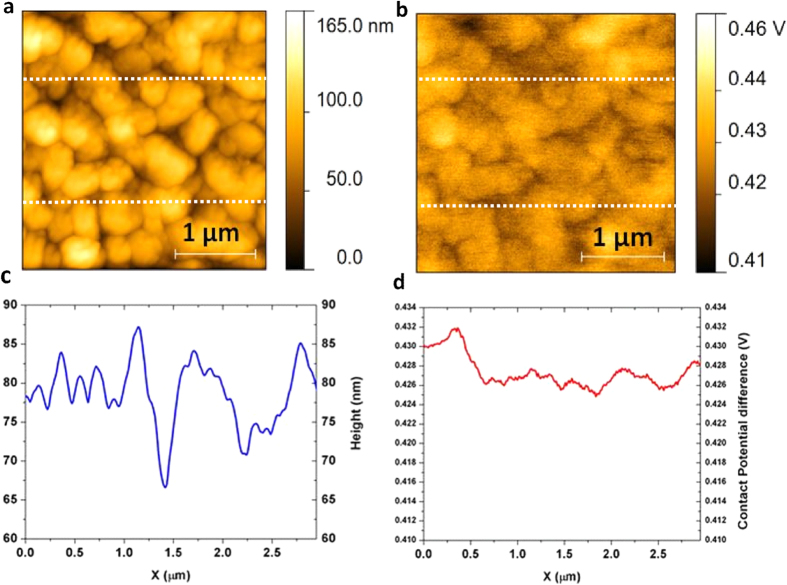
KPFM characterization of epitaxial Cu_3_Ge thin film. **(a)** The topographical image. (**b)** The corresponding surface potential image. (**c**) The band profile derived from the middle region (between two white dashed lines) of (**a)**. (**d**) The band profile derived from the middle region (between two white dashed lines) of (**b)**.

Work function is another fundamental electronic property of a metallic surface, affecting both electron emission through the surface and electronic trajectories near the surface^32^. The local work function of Cu_3_Ge thin film φ_Cu3Ge_ was measured by KPFM. Figure 6a,b show the topographical image and the corresponding surface potential image obtained for epitaxial Cu_3_Ge thin film. Small grains of tens of nanometers can be seen clearly from the topographical image, while an almost uniform distribution of V_CPD_ within each grain was observed from the contact potential image. The “band profiles” derived from the middle regions (between two white dashed lines) of Fig. 6a,b are shown in Fig. 6c,d, demonstrating the height and V_cpd_ variation. The average V_CPD_ for epitaxial Cu_3_Ge thin film was measured to be ~0.43 V. Therefore the work function of Cu_3_Ge thin film is ~(4.47 ± 0.02) eV(see supplementary information, section 4 for calculation), which is between the work functions of n^+^ and p^+^-polysilicon^24^. This value is desirable for epitaxial Cu_3_Ge thin film to be used as a mid-gap gate metal directly over SiO_2_ even at very low temperatures for applications in CMOS devices, because it would require minimal and symmetric channel implants even at linewidths below 0.5 μm^24^. The comparison between surface potential maps of epitaxial Cu_3_Ge thin film and polycrystalline Cu_3_Ge thin film with extra Ge phases is shown in Figure S4 (supplementary information, section 5).

## Conclusions

In this paper, the nanoscale electrical characterizations (including current maps, current-voltage spectra, current line profiles, current histograms and surface potential maps) of epitaxial Cu_3_Ge thin film is reported. Current-line profile analysis reveals that current burst generally occurs at local-maximum-height-gradient positions. This phenomenon is due to the variation of local atomic distances and is proved by HRTEM images. The average resistivity of epitaxial Cu_3_Ge thin film is measured to be ~6 ± 1 μΩ cm, 20% smaller than the average value of 8 ± 2 μΩ cm reported for polycrystalline Cu_3_Ge films. The average work function of epitaxial Cu_3_Ge thin film is measured to be ~4.47 ± 0.02 eV, rendering it a desirable mid-gap gate metal to be used directly over SiO_2_ for applications in CMOS devices. The results here show tantalizing prospect of epitaxial Cu_3_Ge thin film as a competitive candidate for next-generation interconnection materials. The results reported here can be used as future reference when epitaxial Cu_3_Ge films on other substrates are studied.

## Methods

### Thin film fabrication

Cu_3_Ge thin film depositions on sapphire substrates were performed by pulsed laser deposition (PLD) at 400 ± 10 °C, with a laser shot frequency of 1HZ. All depositions were carried out in a multi-target stainless steel laser chamber using a pulsed KrF excimer laser (wavelength 248 nm, pulse duration 25 ns). The targets were 4N pure Cu and Ge sheets purchased from ESPI Metals Inc. The PLD chamber was evacuated by a turbo-molecular pump to a vacuum of ~10^−7^ torr. The laser beam was focused onto the targets at a 45° incidence angle and had a square spot size of 2 mm × 3 mm. The laser was excited from its source under “High Voltage Constant” mode so that the exciting voltage was maintained at 23.8 keV (according to previous experience in PLD deposition^33^,^34^,^35^,^36^,^37^,^38^,^39^), and the energy of laser beam at the front of the chamber was ~0.29–0.30 J. As a result, the energy density of the laser beam was estimated to be 4.8–5 J cm^−2^.

### Structural characterization of the as-deposited films

Film structure and orientation were characterized by X-Ray Diffraction (XRD) θ–2θ scan analysis, using a Rigaku X-ray diffractometer with Cu-Ka radiation (λ = 0.154 nm).

Film morphology characterization was performed by FEI Verios 460L high resolution field emission Scanning Electron Microscope (SEM).

Film microstructure was studied by transmission electron microscopy (TEM) using a JEOL-2010F analytical and high-resolution electron microscope equipped with a Gatan image filter tuning attachment, which has a point-to-point resolution of 0.18 nm.

The samples for TEM observation were prepared using Focus Ion Beam (FIB) technique on an FEI Quanta 3D FEG DualBeam instrument. Au and Pt layers were deposited on Cu_3_Ge films during FIB sample preparation for electron conduction and protection.

### Conductive atomic force microscope (CAFM) characterization

Conductive atomic force microscope (CAFM) characterization was carried out by using a Veeco Dimension V AFM, Nanoscope V Controller, and an extended TUNA Module. PtIr coated cantilevers (NSG03/Pt, NT-MDT, Russia, nominal k = 1.7 N/m, f_0_ = 90 kHz) was used for topographic imaging, current mapping and current-voltage measurements. In order to avoid false engagement of the tip, the deflection set-point value of the feedback was adjusted to a higher value (~+300 mV) and the sample bias was set to 0 V before the tip engaged the surface. When the tip contacted the sample surface, the cantilever deflection set-point was adjusted to a smaller value (~+20 mV) to decrease the effects of wearing on the conductive tip during scanning in CAFM measurements. The applied sample bias was +100 mV for current mappings to preclude possible surface modifications and retain Ohmic contact behavior. To avoid damages of the tip coating and to minimize the influence of parasitic capacitance effects at the tip-to sample interface, the scan rate of CAFM mapping was less than 0.5 Hz. The recorded current as a function of position provides a map of the local capacity of the sample to transport charge between the tip position and the Ag counter electrode (silver paste applied on the sample surface). Topographic images and the corresponding current maps of the sample were obtained simultaneously with a scan size of 1.5 μm, and a scan rate of 0.3 Hz. Current-voltage measurements at specific positions on the sample surface were carried out by using the software’s “point and shoot” feature and fixing the conducting tip at appropriate surface structures of interest.

### Bulk resistivity measurement

The Cu_3_Ge film was placed in a Lakeshore probe station (Lake Shore Cryotronics, Inc., Westerville, USA) for non-destructive electrical testing. The versatile and state-of-the-art Lakeshore probe station is equipped with six independently manipulated probe arms (each holding a DC probe), and an optical system (microscope, camera, light source, and monitor) to view the sample under test. Probes were moved into place on the sample while observing with the microscope. Measurements of sample properties were then made via the probes. The input/output electrical signal was controlled by an Agilent 4155C semiconductor parameter analyzer. The noise level of the setup is below 1 pico ampere.

### Kelvin probe force microscopy (KPFM) characterization

In the KPFM measurements, the conductive tip (NSG03/Pt) was working in tapping mode and lift mode scan. Reliable topographic images were obtained before the KPFM measurements. In KPFM tests, an AC and a DC bias were applied on the cantilever while the sample remained grounded. The frequency of the AC signal was set at ~2 kHz lower than the resonance frequency of the cantilever and the amplitude ac voltage was 560 mV. The lift scan height was 50 nm and the scan rate was 0.75 Hz.

All AFM images were analyzed and processed using Gwyddion (open source software version 2.3).

## Additional Information

**How to cite this article**: Wu, F. *et al.* Nanoscale electrical properties of epitaxial Cu_3_Ge film. *Sci. Rep.* **6**, 28818; doi: 10.1038/srep28818 (2016).

## Supplementary Material

Supplementary Information
