# Supplementary material for: Nanoscale electrical properties of epitaxial Cu3Ge film
Source: Sci Rep. 2016 Jul 1;6:28818. doi: 10.1038/srep28818 (PMC4929471; doi:10.1038/srep28818)
Supplement: Supplementary Information [file srep28818-s1.pdf]

Supplementary Information

Nanoscale electrical properties of epitaxial Cu_3Ge film

F. Wu, W. Cai, and N. Yao

Princeton Institute for the Science and Technology of Materials (PRISM), Princeton University, 70 Prospect Avenue, Princeton, New Jersey 08540, USA

Corresponding authors:
Nan Yao: Tel: 609-258-6394;Email: nyao@princeton.edu
Fan Wu: Tel: 919-760-0269; Email: fanwu@princeton.edu

Section 1: Details of PLD deposition for five samples

Section 2: Additional supporting evidence for Current line-scan profile analysis of epitaxial Cu_3Ge thin film.

Section 3: Calculation of resistivity of epitaxial Cu_3Ge thin film

Section 4: KPFM working principle and calculation of work function for epitaxial Cu_3Ge thin film

Section 5: Comparison between surface potential maps and height maps of epitaxial Cu_3Ge thin film and polycrystalline Cu_3Ge thin film with extra Ge phases.

Section 6: Supplementary references

1. **Details of PLD deposition for five samples**

Multiple repetitions of Ge and Cu depositions on sapphire substrates were performed by pulsed laser deposition (PLD). Specifically, five samples were deposited on c-plane sapphire substrates at 400±10 °C, with a laser shot frequency of 1HZ. 90 thin layers of Cu and Ge (thus a total of 180 layers) were deposited alternatively on c-sapphire for each sample. Cu and Ge layers were expected to diffuse and react to form Cu_3Ge thin films because of the excess kinetic energies provided by laser ablation [1-3]. Deposition parameters were varied to systematically investigate their effects on the crystallinity and microstructure of the Cu_3Ge films. The thickness of each Cu and Ge layer remains unchanged for the same sample, but decreased from sample 1 to sample 5, resulting to thinner Cu/Ge layers and higher reaction rates. The laser pulse numbers in each repetition of Cu and Ge ablations for five samples are listed as following: sample 1: Cu:Ge=35:5; sample 2: Cu:Ge=25:5; sample 3: Cu:Ge=15:5; sample 4: Cu:Ge=14:2; sample 5: Cu:Ge=7:1. Therefore the crystallinities of Cu_3Ge films were estimated to improve from sample 1 to sample 5. All depositions were carried out in a multi-target stainless steel laser chamber using a pulsed KrF excimer laser (wavelength 248 nm, pulse duration 25 ns). The targets were 4N pure Cu and Ge sheets purchased from ESPI Metals Inc. The PLD chamber was evacuated by a turbo-molecular pump to a vacuum of ~ 10^{-7} torr. During the heating of the substrate to the deposition temperature (400 ±10 °C), the chamber vacuum decreased to ~ 3.0×10^{-4} torr. The laser beam was focused onto the targets at a 45° incidence angle and had a square spot size of 2mm× 3 mm. The laser was excited from its source under "High Voltage Constant" mode so that the exciting voltage was maintained at 23.8 keV (according to previous experience in PLD deposition [4-12]), and the energy of laser beam at the front of the chamber was ~ 0.29–0.30 J. As a result, the energy density of the laser beam was estimated to be 4.8 - 5 J cm^{-2}. The sapphire substrates were initially cleaned through a multi-step procedure including boiling in acetone at 150 °C for 5 min, ultrasonic cleaning in acetone for 5 min and ultrasonic cleaning in methanol for 5 min. The cleaned substrates were fully dried by nitrogen gun and then loaded into the deposition chamber where the target–substrate distance was 4 cm. The sapphire substrates were held parallel to the

targets during deposition, and the targets were rotated in order to provide a uniform ablation and avoid pitting on the target surface.

2. Additional supporting evidence for Current line-scan profile analysis of epitaxial Cu_3Ge thin film.

To show that the relationship between the localized electron transport capability and film morphology is ubiquitously true instead of a coincidence, line-scan profile analyses were performed on the top, middle and bottom region of the morphological (Figure 2b) and current maps (Figure 2e). Figure S1 - S3 show the typical line profiles derived from the three regions, supporting that current burst generally occurs at a position where height change rate reaches a local maximum. These supplementary figures, together with Figure 3, demonstrate that the correlation between current burst and local maximum height change rate is a common phenomenon for the epitaxial Cu_3Ge thin film.

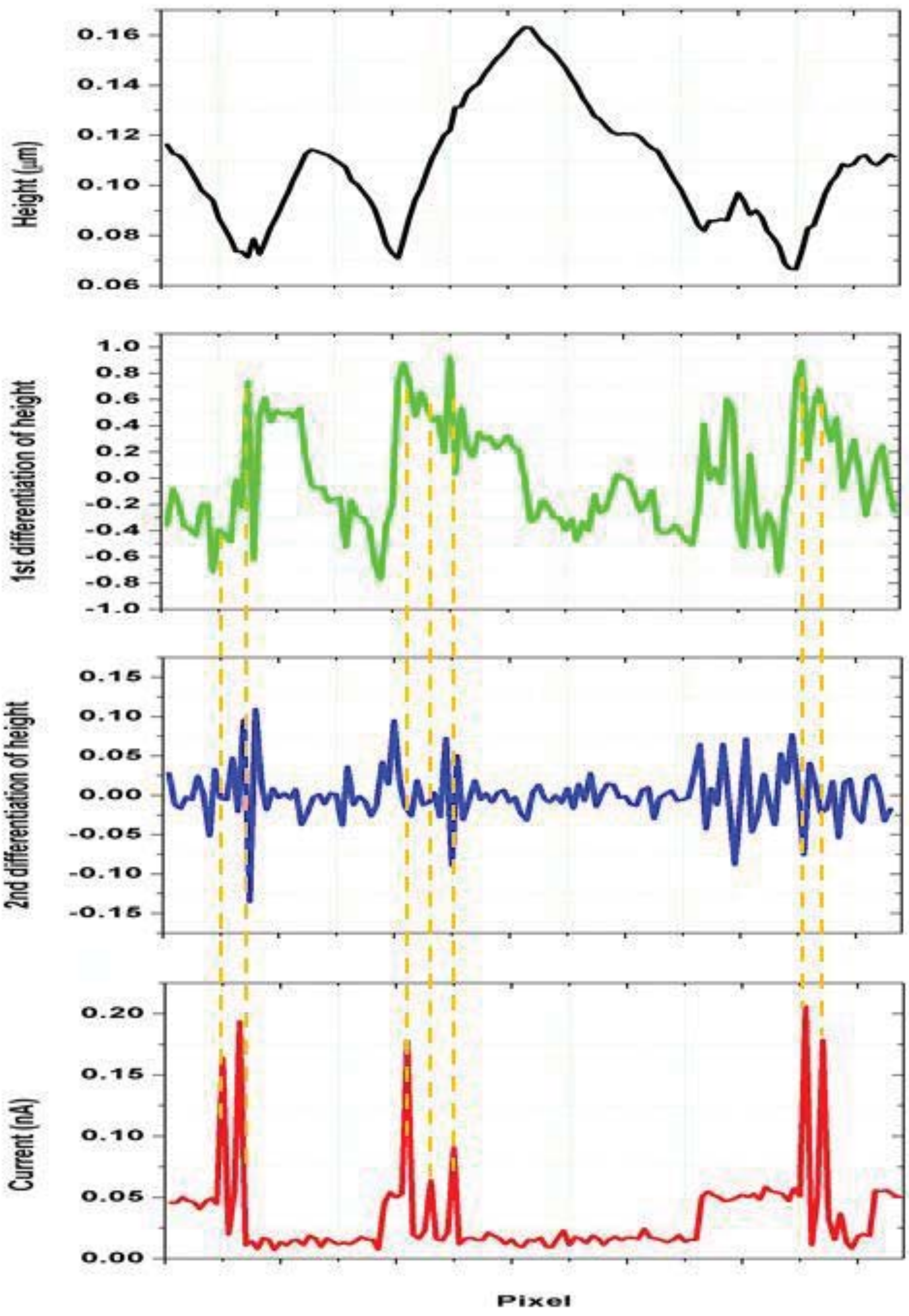

Figure S1| Current line-scan profiles derived from the top region of the height image (Figure 2b) and the current map (Figure 2e).

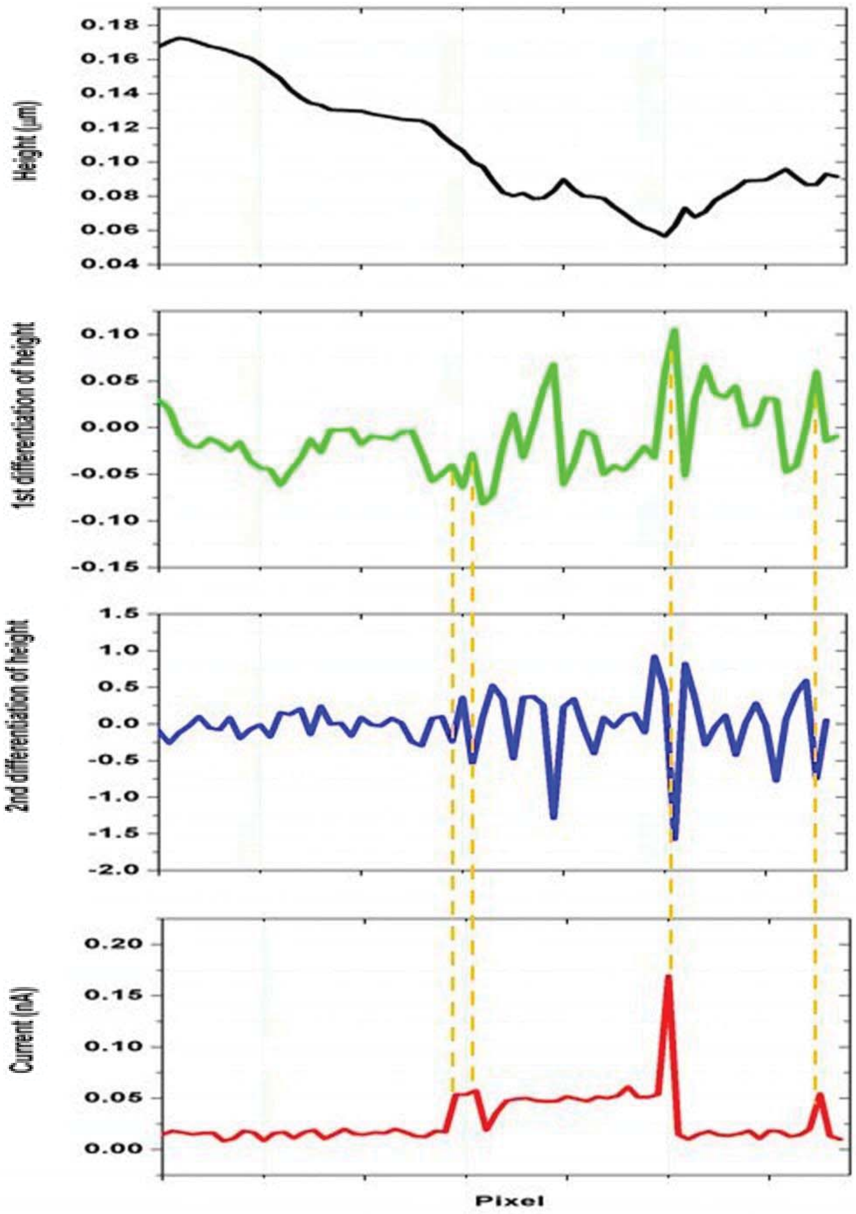

Figure S2| Current line-scan profiles derived from the middle region of the height image (Figure 2b) and the current map (Figure 2e).

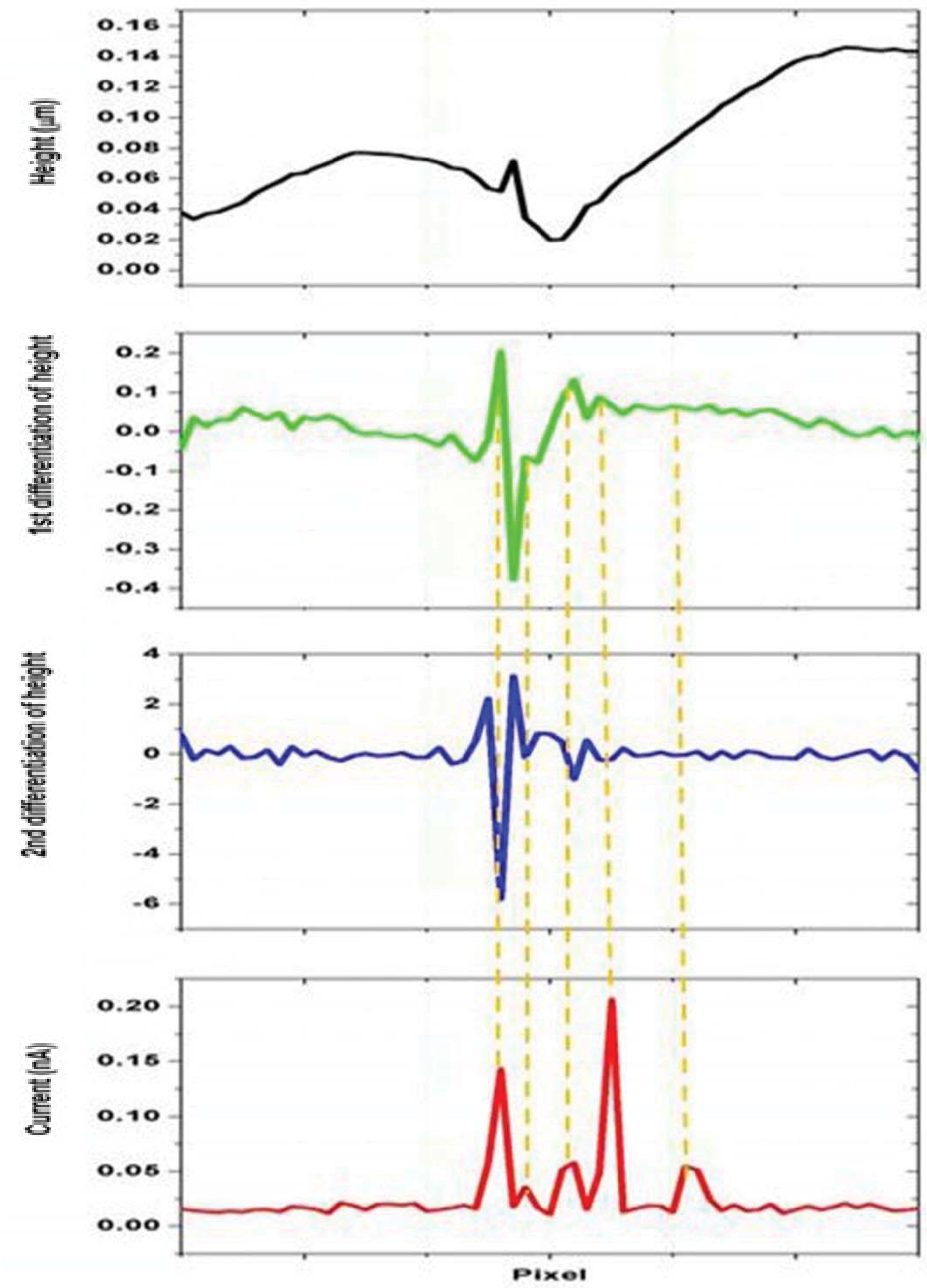

Figure S3| Current line-scan profiles derived from the bottom region of the height image (Figure 2b) and the current map (Figure 2e).

3. Calculation of resistivity of epitaxial Cu_3Ge thin film

According to the Hertzian contact mechanics model, the radius of the contact area between a tip and the sample is given by [13]:

$$r_c = \sqrt[3]{\frac{\mathrm{F\ r}_t}{E_r}}$$

Where r_c is the radius of the contact area, F is the force between the tip and the sample, r_t is the tip radius and E_r is the elastic modulus of Cu_3Ge thin film [13]. r_t is ~25 $\pm$ 5 nm and F is ~1 nN (the product of the deflection error sensitivity η, the spring constant k of the Pt/Ir tip, and the applied voltage V). The elastic modulus of Cu_3Ge thin film was measured by nanoindentation [14]:

$$\frac{1}{E_r} = 1 - \frac{\nu_{indentor}^2}{E_{indentor}} + 1 - \frac{\nu_{Cu_3Ge}^2}{E_{Cu_3Ge}}$$

Where ν is Poisson's ratio for the indenter and sample, and E is the respective elastic moduli. The obtained elastic modulus was 112 GPa for room-temperature Cu_3Ge [14] film and is adopted for this study. Therefore r_c is calculated to be ~0.5 $\pm$ 0.1 nm, and the cross-sectional area (A) for current to go through is r_c^2, on the order of 0.25 nm^2. Consequently the average resistivity of epitaxial Cu_3Ge thin film can be calculated as:

$$\rho = \frac{A \times t\text{he slope of the fitted line in Figure 4f}}{1\mu m} \approx 6 \pm 1\ \mu\Omega\ \mathrm{cm}$$

Which is 20% smaller than the average value (8 $\pm$ 2 μΩcm) reported for polycrystalline Cu_3Ge films [14,15]. Considering that diffusion barrier is no longer needed for Cu_3Ge to replace Cu, the overall resistivity of epitaxial Cu_3Ge thin film qualifies its application as the new-generation interconnection material.

4. KPFM working principle and calculation of work function for epitaxial Cu_3Ge thin film

KPFM measures the work function of solid surfaces at atomic or molecular scales, demonstrating information about composition and electronic state of the local structures on the surface. When the conducting tip and the sample are brought in contact, a net electric current would flow between them until the Fermi levels were aligned. During measurement a voltage is applied between tip and sample, consisting of a DC-bias and an AC-voltage:

$$V = (V_{DC} - V_{CPD}) + V_{AC} sin(\omega t)$$

Where V_{CPD} is the contact potential difference between a conductive tip and a sample, $V_{AC}sin(\omega t)$ and V_{DC} are the applied AC voltage of frequency ω and DC voltage on the tip. The electrostatic force in a capacitor can be written as:

$$F = \frac{1}{2}\frac{dC}{dz}V^2$$

Where C is the tip-sample capacitance, z is the tip-sample distance, and V is the tip-sample voltage. Combining the above two equations, the electrostatic force can be split up into three contributions:

$$F = F_{DC} + F_{\omega} + F_{2\omega}$$

The DC component (F_{DC}) contributes to the topographical signal, the term F_{ω} measures the contact potential and the contribution $F_{2\omega}$ can be used for capacitance microscopy. They can be expressed as following:

$$F_{dc} = \frac{dC}{dz}\,[\frac{1}{2}(V_{DC} - V_{CPD})^2 + \frac{1}{4}\,V_{AC}^2]$$

$$F_{\omega} = -\frac{dC}{dz}\,(V_{DC} - V_{CPD})V_{AC} sin(\omega t)$$

$$F_{2\omega} = -\frac{1}{4}\frac{dC}{dz}\,V_{AC}^2 cos(2\omega t)$$

When V_{DC}= V_{CPD}, the electrostatic force component measured at frequency ω and the oscillating amplitude would be zero. Consequently V_{DC} can track V_{CPD} at each point of the scan area by using a feedback circuit. Once V_{CPD} is obtained, the local work function of Cu_3Ge thin film φ_{Cu_3Ge} can be calculated as:

$$eV_{CPD} = \varphi_{tip} - \varphi_{Cu_3Ge}$$

Where φ_{tip} is the work function of the conductive tip (~4.90 $\pm$ 0.02 eV)[31], calibrated with a freshly cleaved highly oriented pyrolytic graphite (HOPG, Grade 1 SPI, 4.65 eV)[31] before the experiment.

The average V_{CPD} for epitaxial Cu_3Ge thin film was measured to be ~0.43 V from Figure 5d. Therefore the work function of Cu_3Ge thin film is

$$\varphi_{Cu_3Ge} = \varphi_{tip} - eV_{CPD} = (4.9 \pm 0.02) - 0.43 = (4.47 \pm 0.02)eV$$

5. Comparison between surface potential maps and height maps of epitaxial Cu_3Ge thin film and polycrystalline Cu_3Ge thin film with extra Ge phases.

To understand the relationship between surface potential and film morphology/structure, the surface potential maps of epitaxial Cu_3Ge thin film and polycrystalline Cu_3Ge thin film with extra Ge phases are compared, as shown in Figure S4. Figure S4a and b are the height maps of epitaxial Cu_3Ge thin film and polycrystalline Cu_3Ge thin film with extra Ge phases, respectively. Their corresponding surface potential maps are shown in Figure S4c and d. From the height maps alone, one can hardly tell the difference among individual islands in two samples and it's difficult to distinguish Cu_3Ge phase from Ge phase. In contrast, the difference between surface potentials of two samples is obvious. For epitaxial Cu_3Ge thin film, the surface potential distribution is almost uniform. The deviation from the maximum to the minimum surface potential is only 0.084v, and the minimum surface potential only appears in the grain boundary region, which indicates that the islands are composed of the same phase. Nevertheless for the polycrystalline Cu_3Ge thin film with extra Ge phases, the deviation from the maximum to the minimum surface potentials is 0.19v, which is 250% of

that for the epitaxial Cu_3Ge film. More importantly, large regions of low-potential area can be distinguished from high-potential area, indicating that the individual islands are composed of different phases, regardless of the similar morphological appearance.

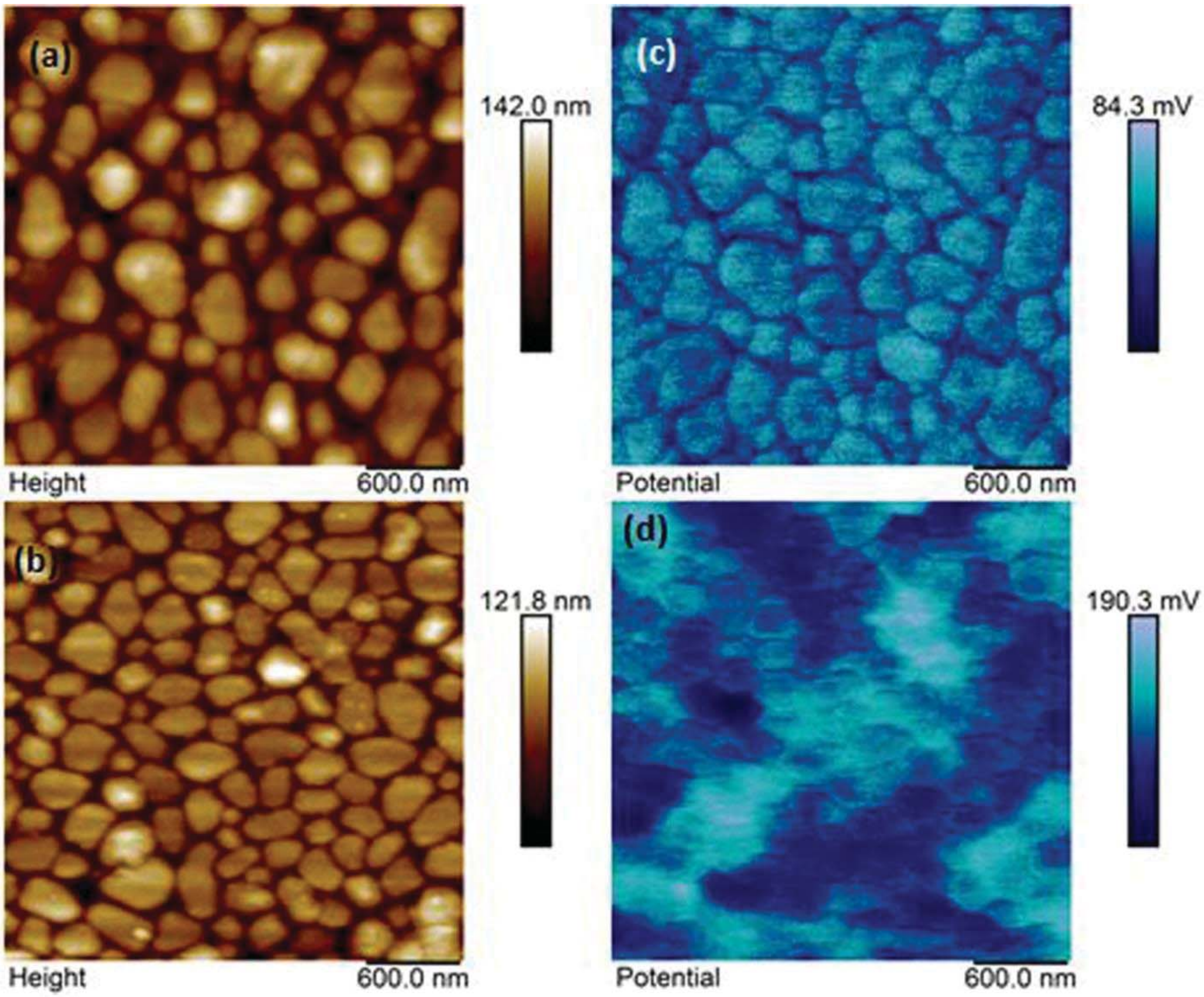

Figure S4| Comparison between surface potential maps and height maps of epitaxial Cu_3Ge thin film and polycrystalline Cu_3Ge thin film with extra Ge phases. a. The height map of epitaxial Cu_3Ge thin film. **b.** The height map of polycrystalline Cu_3Ge thin film with extra Ge phases. **c.** The corresponding surface potential map of epitaxial Cu_3Ge thin film. **d.** The corresponding surface potential map of polycrystalline Cu_3Ge thin film with extra Ge phases.

6. Supplementary references

1 Wu, F., Rao, S. S., Prater, J. T., Zhu, Y. T. & Narayan, J. Tuning exchange bias in epitaxial Ni/MgO/TiN heterostructures integrated on Si(100). *Current Opinion in Solid State and Materials Science* **18**, 263-268, (2014).

2 Wu, F. & Narayan, J. Controlled epitaxial growth of body-centered cubic and face-centered cubic Cu on MgO for integration on Si. *Crystal Growth & Design* **13**, 5018-5024, (2013).

3 Wu, F. *Planar defects in metallic thin film heterostructures*, North Carolina State University, (2014).

4 Rao, S. S. *et al.* Positive exchange bias in epitaxial permalloy/MgO integrated with Si (100). *Current Opinion in Solid State and Materials Science* **18**, 140-146, (2014).

5 Rao, S. S. *et al.* Interface magnetism in epitaxial $BiFeO_3$-$La_{0.7}Sr_{0.3}MnO_3$ heterostructures integrated on Si (100). *Nano letters* **13**, 5814-5821 (2013).

6 Lee, Y. *et al.* Epitaxial integration of dilute magnetic semiconductor Sr_3SnO with Si (001). *Applied Physics Letters* **103**, 112101 (2013).

7 Bayati, M. *et al.* Correlation between structure and semiconductor-to-metal transition characteristics of VO_2TiO_2/sapphire thin film heterostructures. *Acta Materialia* **61**, 7805-7815 (2013).

8 Molaei, R., Bayati, R., Wu, F. & Narayan, J. A microstructural approach toward the effect of thickness on semiconductor-to-metal transition characteristics of VO_2 epilayers. *Journal of Applied Physics* **115**, 164311 (2014).

9 Lee, Y., Wu, F., Narayan, J. & Schwartz, J. Oxygen vacancy enhanced room-temperature ferromagnetism in Sr_3SnO/c-YSZ/Si (001) heterostructures. *MRS Communications* **4**, 7-13 (2014).

10 Rao, S. *et al.* Integration of epitaxial permalloy on Si (100) through domain matching epitaxy paradigm. *Current Opinion in Solid State and Materials Science* **18**, 1–5 (2013).

11 Singamaneni, S. R., Prater, J., Wu, F. & Narayan, J. Interface magnetism of two functional epitaxial ferromagnetic oxides integrated with Si (100). *Bulletin of the American Physical Society* (2014).

12 Singamaneni, S. R. *et al.* Positive exchange bias in epitaxial permalloy/MgO integrated with Si (100). *Bulletin of the American Physical Society* (2014).

13 Mativetsky, J. M. *et al.* Local Current Mapping and Patterning of Reduced Graphene Oxide. *Journal of the American Chemical Society* **132**, 14130-14136, (2010).

14 Darling, K. A. *et al.* Thermal stability, mechanical and electrical properties of nanocrystalline Cu_3Ge. *Intermetallics* **16**, 378-383, (2008).

15 Tawancy, H. M. & Aboelfotoh, M. O. Effect of phase transitions in copper-germanium thin film alloys on their electrical resistivity. *J Mater Sci* **30**, 6053-6064, (1995).
